# HLA class I anchor motifs contribute to antigen presentation from non-canonical open reading frames

**DOI:** 10.3389/fimmu.2026.1939526

**Published:** 2026-10-22

**Authors:** Andrea Rivera, Elena Lorente, Daniel López

**Affiliations:** Presentation and Immune Regulation Unit, Centro Nacional de Microbiología, Instituto de Salud Carlos III, Majadahonda, Spain

**Keywords:** anchor motif, antigen processing, cancer, HLA, mass spectrometry, ncORFs

## Abstract

Non-canonical open reading frames (ncORFs) encoded throughout the human genome can be translated into unconventional proteins, several of which are implicated in tumorigenesis through disruption of key regulatory pathways. Although previous studies showed that ncORF-derived peptides are presented by human leukocyte antigen (HLA) class I molecules, their overall contribution to the HLA class I immunopeptidome relative to the canonical proteome remains unclear. We used a database comprising 7, 264 human ncORFs sequences to identify ncORF-derived peptide ligands in mass spectrometry datasets covering 102 HLA class I classical and non-classical alleles to determine their role as parental proteins of HLA class I ligands compared to the canonical proteome. Across these alleles, covering the vast majority of the human population, ncORF-derived ligands represented an average of 2.13% of the total HLA class I ligandome although their representation varied substantially between individual alleles. Notably, HLA class I molecules with Alanine, Proline, or basic as anchor residues displayed a higher proportion of these ncORF-derived peptides, and the C-terminal peptide anchor motif showed the strongest association with ncORF-derived ligand presentation. Likewise, ncORF-derived proteins containing HLA class I-bound epitopes exhibited higher transcript abundance, were more basic and less hydrophilic compared to ncORFs that did not generate class I HLA ligands. Overall, ncORF-derived peptides represented a fraction of the HLA class I ligandome and revealed HLA allele-specific anchor motif preferences shape immunopeptidomic representation. Our results expand the current understanding of the antigenic repertoire landscape of healthy organs and, above all, tumor tissues.

## 1 Introduction

Tumors develop from uncontrolled cell proliferation in the body and are one of the world’s leading causes of death. Various therapeutic approaches are currently being used to control or eliminate cancer cells. However, oncology patients may experience severe toxic side effects and drug resistance, which reduces the effectiveness of these strategies. Consequently, identifying novel molecular targets involved in cancer progression and antitumor immune responses is essential for the development of more effective cancer therapies.

Ribosome profiling (Ribo-seq) is a novel technique that has substantially expanded our understanding of the translational landscape of the human genome. This powerful approach has revealed thousands of previously unannotated non-canonical open reading frames (ncORFs), many of which originate from genomic regions that were historically classified as non-coding during the Human Genome Project owing to the absence of canonical translation signatures.

Subsequently, mass spectrometry (MS) analysis was performed to confirm the expression of these ncORF-derived proteins. However, conventional whole-proteome MS approaches detect only a very small fraction of ncORF-derived protein products, typically ranging from nearly undetectable levels to just 0.1-0.2% of all cellular tryptic peptides identified (1, 2). One reason is that some ncORFs are located within GC-rich regions and encode arginine-rich amino acid sequences (CGA, CGC, CGG and CGU codons), rendering them highly susceptible to trypsin cleavage and generating peptides that are too short for efficient MS detection. In addition, these non-canonical proteins are generally small, expressed at low levels (1) and are more related to degradation than canonical proteins (3).

In contrast, MS-based immunopeptidomics has demonstrated expression of non-canonical peptides presented by human leukocyte antigen (HLA) class I molecules originate from genuinely translated proteins derived from these “dark proteome” regions protein products with the ability to perform multiple functions within the cell (4). These specific immunopeptidome analyses revealed that ncORF-derived peptides represent approximately 1–4% of the total identified HLA-associated peptide repertoire, depending on the biological sample analyzed (2). This frequency is approximately 10- to 40-fold higher than the detection range of whole proteome experiments (2) probably because of the rapid degradation of these ncORF-derived protein products might look like rapid antigen processing resembling defective ribosomal products (DRiPs), the major source of HLA ligands (5).

Interestingly, many ncORFs encode functional microproteins involved in key regulatory pathways, such as those responsible for cell proliferation, apoptosis, migration, invasion, angiogenesis, inflammation, DNA damage responses and genomic instability (reviewed in (6)). Dysregulation of these pathways contributes to malignant transformation and tumour progression. In this context, HLA class I peptide ligands derived from translated ncORFs may be important from an immunological standpoint, since these unconventional proteins are sometimes specifically expressed in various types of tumor tissues (7). Therefore, ncORF-derived peptides may represent promising targets for next-generation cancer immunotherapies.

However, the contribution of ncORF-derived proteins to the HLA class I ligandome relative to canonical proteins remains poorly defined, particularly in the context of HLA polymorphisms. To address this question, we have re-analyzed previously published MS datasets comprising 102 classical and non-classical HLA class I alleles, which are widely distributed across the human population, to systematically assess the contribution of ncORF-derived proteins to HLA class I immunopeptidome across diverse HLA alleles.

## 2 Material and methods

### 2.1 Database searches and mass spectrometry data processing

A comprehensive literature search was conducted to identify mass spectrometry (MS)-based studies on HLA class I ligands. Only studies with an unambiguous assignment of HLA class I allele at the subtype level were selected. Raw MS datasets from the public proteomics repository MassIVE: MSV000084172 (8), MSV000095860 (9) and the PRIDE repository with the ProteomeXchange accession: PXD023064 (10), PXD007679 (11), PXD006215 (12), PXD014804 (13), PXD020186 (14), PXD035241 (15), and PXD009531 (16) were selected. Across these nine studies, B-lymphoblastoid HLA class I-deficient cell lines were employed in seven datasets (8, 9, 11–14, 16), while an erythroleukemic cell line (10) and a lung adenocarcinoma cell line (15) were used in the remaining two. The raw mass spectrometry data included experiments, with 102 distinct HLA class I alleles processed using Peaks Studio v12.5 (Bioinformatics Solutions Inc., Waterloo, Canada).

Raw MS/MS spectra were analyzed using Peaks Studio v12.5 (Bioinformatics Solutions Inc., Waterloo, Canada). Searches were performed using a combined database comprising the human UniProt/Swiss-Prot database (February 4, 2025; 20, 412 proteins) and a curated database of 7, 264 translated ncORFs (17). The search was not limited to enzymatic specificity. The length of the peptides ranged from 8 to 25. Peptide mass and the fragment ion tolerances were set at 0.1 and 0.2 Da, respectively. The identified peptides for each HLA class allele were selected when their -10logP scores from Peaks 12.5 were ≥ 15. Additionally, a strict 1% false discovery rate (FDR) was established to search against a database with the reversed human sequences. To resolve ambiguous peptide assignments and prevent false-positive ncORF-derived peptide identifications, a strict requirement of at least 1 unique peptide per entry (Protein Unique Peptides ≥ 1) was enforced. Oxidation of methionine (M) was used as a variable modification.

Following PEAKS analyses, data peptide sequences of each allele were manually curated to resolve shared sequence ambiguities. Any peptide sharing total sequence identity between a canonical human protein and a ncORF-encoded protein was strictly assigned to the canonical proteome and excluded from the translated ncORF dataset.

### 2.2 Functional bioinformatics procedures

HLA class I peptide-binding affinities for each identified peptide were predicted against the specific HLA allele, corresponding to its respective MS run, using NetMHCpan 4.1 (18). Weak or non-binding peptides with a % rank of ≥ 10% were excluded. For redundant epitopes sharing the same allele, only the one with the highest score was considered. Based on these analyses, the ratio between ncORF-derived HLA class I ligands and the standard proteome was calculated.

Peptide-binding anchor motifs recognized by HLA-A, -B, -C, -E and -G molecules were compiled from our own MS analyzed. Specific anchor preference was assigned to an HLA allele when a particular amino acid or physicochemical family constituted the vast majority (e.g., >80%) of the observed residues at that anchor position. These experimentally derived positional preferences were subsequently confirmed and validated against consensus anchor motifs reported in the SYFPEITHI database (19). Finally, HLA class I alleles were categorized based on the dominant physicochemical properties of their validated anchor residues (basic, acidic, aromatic, hydrophobic, polar, or proline) and supertype classifications (20).

RPKM (Reads Per Kilobase Million) or TPM (Transcripts Per Million) values used to quantify mRNA expression abundance were collected from previous human Ribo-seq studies (21–26).

The isoelectric point (pI) and GRAVY index were calculated using the Sequence Analysis tool of Sequence Manipulation Suite JavaScript 1.5 (27). The GRAVY index was based on the Kyte and Doolittle hydrophobicity scale (28).

The data of population coverage were calculated with the tool from the IEDB Analysis Resource dataset selecting the different HLA class I molecules with the option “world population”.

### 2.3 Statistical analyses

To analyze the statistical significance of the assays, GraphPad Prism (10.6.1 version) was used. Unpaired Student’s t-tests with Welch’s test were used for comparative analyses between peptides from the standard proteome and ncORF-derived peptides. For the remaining analyses, a nonparametric One-way ANOVA test was used to compare the mean of each column with the mean of every other column and corrected for multiple comparisons with the two-stage step-up method of Benjamini, Krieger and Yekutieli with a desired False Discovery Rate of 0.05. *Q* values were considered to be statistically significant as follows: q < 0.05, *; q < 0.01, **; q < 0.001, ***; and q < 0.0001, ****. In addition, a multiple linear regression analysis including anchor motif class of each HLA class I allele, HLA locus, supertype, original dataset source, and total peptide number by each HLA allele and source was also carried out. To control for protein length as a potential confounding factor, wherein longer ncORF-derived protein inherently yield more potential HLA-binding peptides and thus a higher baseline probability of ligand detection, a multivariable logistic regression analysis was performed using the following parameters: the ncORF-derived proteins that generates HLA class I ligands as dependent variable; as independent variable, the length of ncORF-derived proteins; and RPKM, pI, and hydrophobicity as covariates.

## 3 Results

### 3.1 Comparison of HLA class I ligandome derived from the standard proteome and 7, 264 ncORFs across 102 HLA class I alleles

Using the Peaks bioinformatics platform, the MS datasets of the HLA class I ligandome from 102 alleles reported in the literature were reanalyzed against a human protein database comprising both the standard proteome and 7, 264 ncORFs with strong evidence of their translation (17). This analysis assigned 194, 362 fragmentation spectra to cellular peptide sequences associated with different HLA class I classical and non- classical molecules. 70, 796, 90, 314 and 29, 103 ligands were bound to HLA-A, -B and -C, respectively (**Supplementary Table 1**). In addition, other 4, 149 fragmentation spectra were identified as peptide ligands bound to HLA-E, and HLA-G molecules. Remarkably, the other 2, 190 fragmentation spectra were assigned as peptide ligands derived from translated ncORFs as follows: 1, 014 presented by HLA-A class I molecules, 912 bound to HLA-B proteins, 218 complexed with HLA-C molecules, and 46 associated with HLA-E and-G molecules (**Supplementary Table 1**). These findings demonstrate that translated ncORFs contribute a discrete but consistent fraction of the repertoire of peptides presented by both classical and non-classical HLA class I molecules.

### 3.2 Peptides derived from translated ncORFs exhibit predicted HLA binding affinities comparable to those of canonical proteome-derived peptides

To assess the potential immunological relevance of ncORF-derived peptides, the binding affinities of HLA ligands originating from translated ncORFs were compared to those of ligands derived from the canonical human proteome. Computational predictions revealed similar distributions of binding affinities for both peptide groups across their corresponding HLA class I molecules, with no statistically significant differences (**Supplementary Figure 1**). These results indicate that HLA class I ligands derived from ncORF-encoded proteins are true ligands for HLA class I classical and non-classical molecules.

### 3.3 Differential contribution of HLA class I polymorphism to the non-conventional HLA class I ligandome

The MS datasets included in this study were obtained by different research groups over several years, using diverse experimental workflows and mass spectrometers with different levels of accuracy and sensitivity. Thus, to normalize these data and to compare the different experiments and alleles, the ratio between HLA class I ligands derived from ncORFs and those from the standard proteome was calculated for each combination of alleles and experiments. For the HLA alleles included in multiple independent studies, this ratio was calculated individually for each experiment. In these cases, the % of HLA ligands calculated as ncORFs/standard proteome was very similar. For example, using the experiments from (8) and (10), the ncORFs/standard ratio was 0.76% for HLA-A*02:01 in both datasets, and 1.86% and 1.80% for HLA-B*58:01, respectively. For HLA-B*40:02, the ratio was 0.53% using (8) data and 0.59% using (13) experiment. For HLA-A*29:02 this ratio was calculated using three 3 datasets: 0.85% (11), 0.71% (10) and 0.68% (8). Therefore, for alleles with multiple datasets, the final ratio was calculated based on the non-redundant peptides from all individual experiments. These results demonstrate the robustness and reproducibility of ncORF-derived ligand frequencies across independent immunopeptidomic datasets.

As shown in **Figure 1** and **Supplementary Table 1**, the contribution of ncORF-derived peptides to the HLA class I ligandome varied substantially among HLA class I alleles. Within the HLA-A locus, the proportion of ncORF-derived ligands ranges from 0.20% to 3.80%. Comparable variability was observed for HLA-B alleles (0.18%-4.52%), HLA-C (0.12%-1.58%), and non-classical HLA class I molecules (0.23%-1.55%). Despite this intra-locus heterogeneity, the average proportion of ncORF-derived ligands was significantly higher for HLAA alleles (1.5 ± 1.1%) than for HLA-B alleles (1.1 ± 1.1%) and, above all, than in HLA-C alleles (0.7 ± 0.4%, **Figure 1**; **Supplementary Table 1**). Considering all 102 HLA class I alleles analyzed, the overall mean contribution of ncORF-derived peptides was 1.1 ± 0.9%.

**Figure 1:**
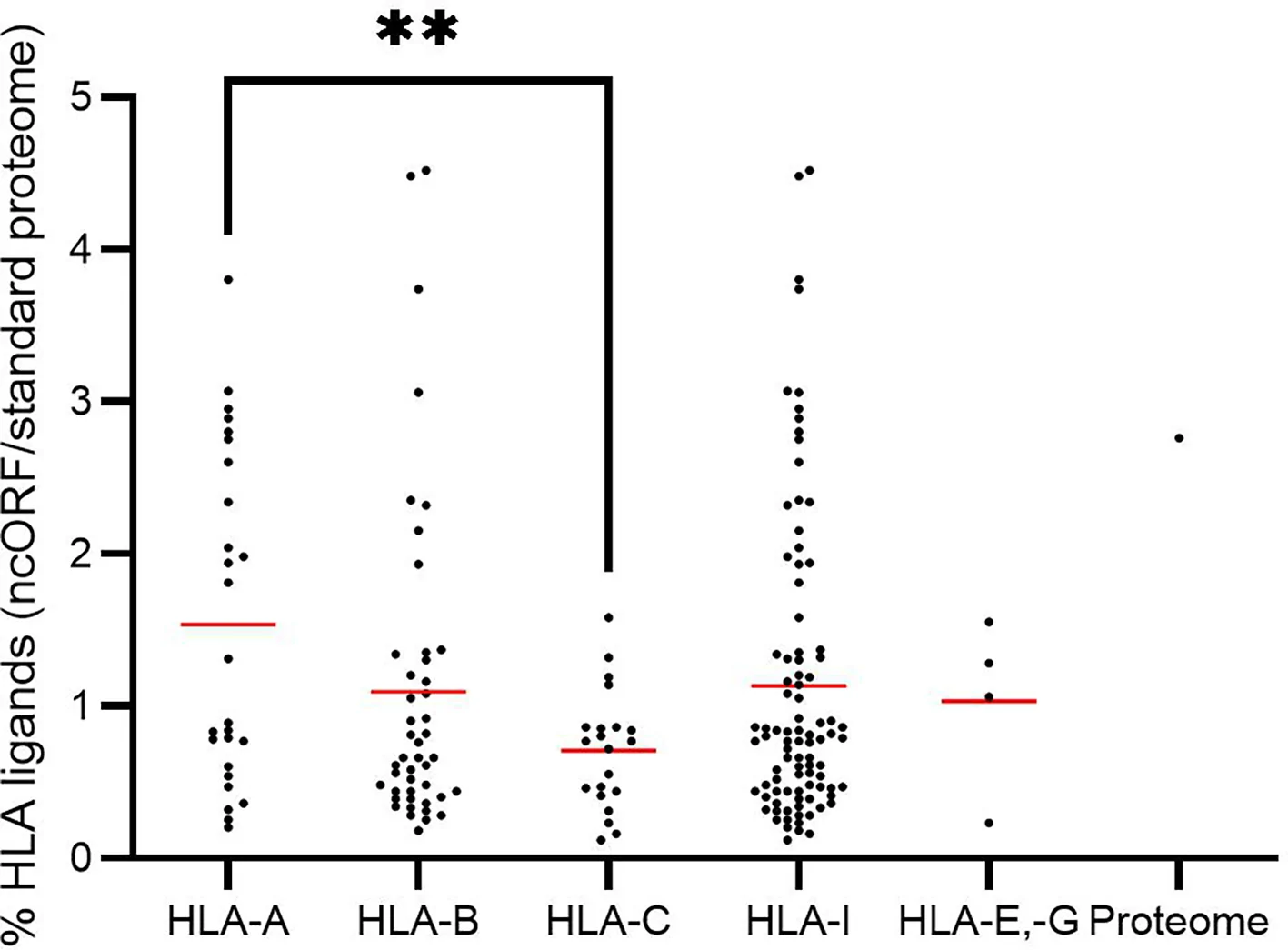
Proportion of HLA class I ligands identified in the 7, 264 ncORFs compared to the standard proteome by HLA class I allele. Percentage of HLA class I peptide ligands (ncORFs/standard proteome) presented by each of the 102 HLA class I molecules included in this study, which cover > 90% global population coverage according to (20) and recalculated using the IEDB Population Coverage Tool (IEDB Analysis Resource) with average worldwide allele frequencies, grouped by HLA loci and total class I molecules. In addition, the ratio between the number of amino acids coded in the translated ncORFs and the standard proteome is indicated. Statistical significance is shown as follows: p < 0.01, **.

On the other hand, the 7, 264 translated ncORFs included in this study encoded a total of 314, 984 amino acid residues, representing only 2.76% of the sequence space of the canonical human proteome. Thus, the proportion of ncORF-derived peptides detected in the HLA class I ligandome corresponds to approximately 40% of the relative percentage predicted strictly from their contribution to total proteome size. Rather than reflecting an intrinsic biological selection against ncORFs, this lower relative yield in the ligandome aligns with the study by Wacholder et al., which showed that the low detection rate of ncORF products can be largely explained by their small size and low translation levels, and does not indicate that they are unstable or biologically insignificant (1). Alternatively, canonical and non-canonical proteins may differ in terms of expression, translation efficiency, degradation, peptide generation, HLA binding, and mass-spectrometric detectability. All these characteristics could explain their reduced detection frequency in the total HLA class I ligandome.

Nevertheless, this pattern of presentation is not uniform across HLA class I polymorphisms. Notably, there are five HLA-A alleles and another four HLA-B molecules that have a percentage of HLA ligands derived from ncORFs greater than 2.8%. These observations suggest that the contribution of translated ncORFs to cellular immunopeptidomes varies markedly across distinct HLA polymorphisms.

### 3.4 Transcript abundance and physicochemical properties influence the contribution of ncORFs to the HLA class I ligandome

To identify the factors associated with the presentation of ncORF-derived peptides by HLA class I molecules, we compared the characteristics of ncORFs that generated HLA ligands with those that did not. Previous analyses of 7, 264 ncORFs included estimates of transcript abundance obtained by RNA sequencing (RNA-Seq), which, despite its limitations, is frequently used as a proxy for protein expression levels (29). Using this data, we found that transcripts harbouring ncORFs that produced HLA class I ligands were statistically more abundant than those from other ncORFs that did not generate HLA-bound peptides (**Figures 2A, B**). These results suggest that transcript abundance is highly correlated with antigen processing and presentation in the non-canonical proteome.

**Figure 2:**
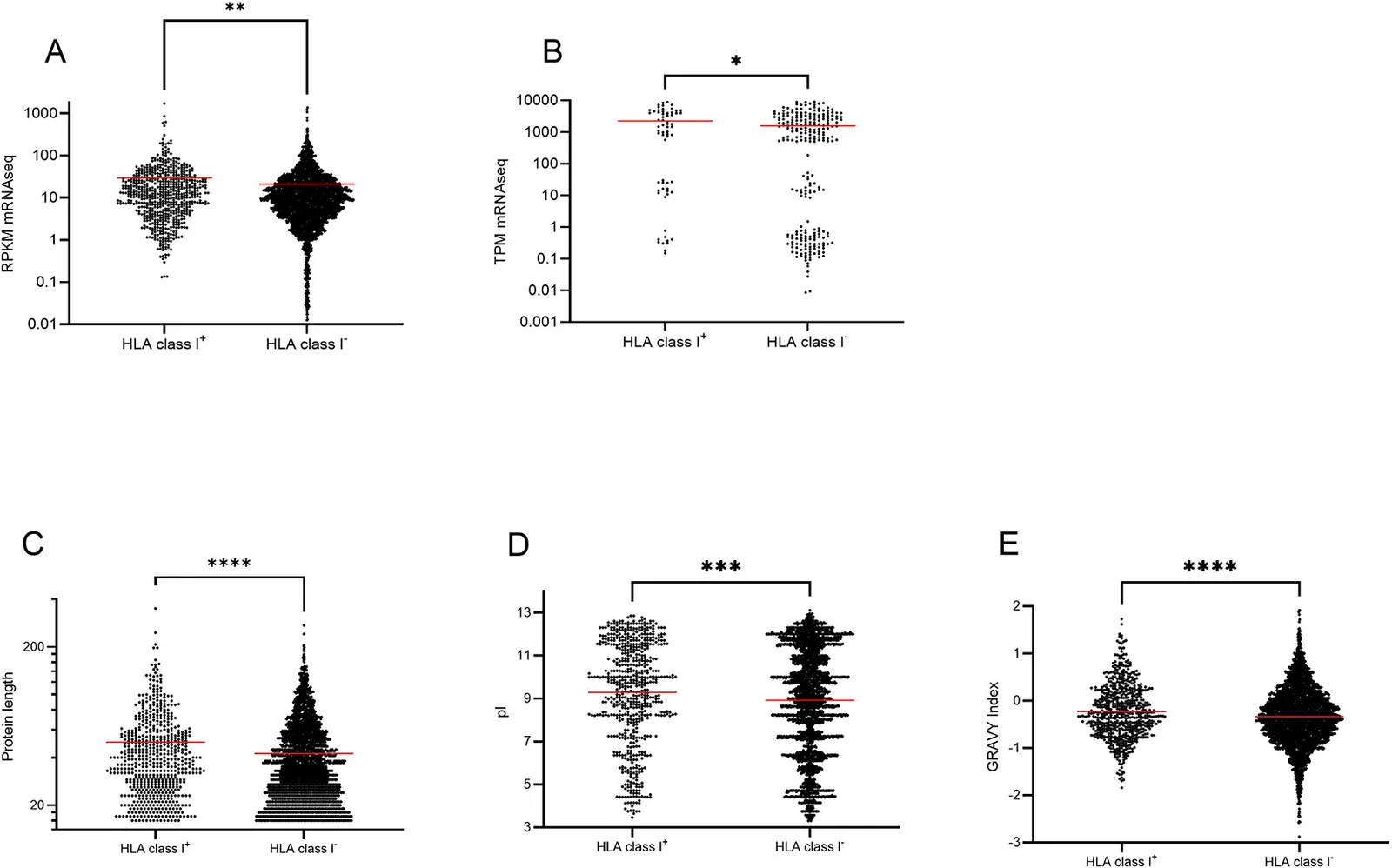
Ribo-seq, length, isoelectric point (pI) and hydrophobicity analysis of parental and non-parental ncORFs proteins for HLA class I ligands. RPKM **(A)**, TPM **(B)** mRNAseq, length **(C)**, pI **(D)** and hydrophobicity were measured as GRAVY index **(E)** of the parental (HLA class I^+^) and non-parental (HLA class I^-^) ncORFs proteins for HLA class I ligands. Statistical significance is shown as follows: p < 0.05, *; p < 0.01, **; p < 0.001, ***; and p < 0.0001, ****.

Next, we investigated whether additional protein features could influence the likelihood of ncORF-derived peptide presentation. Comparative analysis of the physicochemical properties of the parental proteins revealed significant differences between ncORFs that contributed to the HLA class I ligandome and those that did not. Parental ncORF proteins for HLA class I ligands were significantly longer, displayed a higher isoelectric point and were less hydrophilic than the non-parental ncORF proteome (**Figures 2C–E**). As longer proteins intrinsically contain more possible peptide sequences and are therefore more likely to generate at least one detectable HLA ligand, the protein length was analyzed as a potential confounding factor in HLA class I antigen presentation. A logistic regression was carried out, showing an estimated effect on antigen presentation odds of 0.5% per additional amino acid (OR = 1.005; 95%CI= 1.003 to 1.008). This model is valid, as the Hosmer-Lemeshow hypothesis test yields a p-value of 0.1859.

These findings indicate that, in addition to expression levels, intrinsic protein characteristics contribute to the probability that translated ncORFs will generate peptides presented by HLA class I molecules.

Together, these results demonstrate that the contribution of translated ncORFs to the HLA class I ligandome is not random but is influenced by both the abundance (as measured by the imperfect approximation of RNA-seq) and physicochemical properties of the source proteins.

### 3.5 A03 and B58 HLA-supertypes display an increased representation of ncORF-derived ligands

To date, the extensive polymorphism of HLA class I molecules has resulted in the identification of more than 30, 000 alleles. However, this astonishing number of molecules can be grouped into three hierarchical levels: families, superfamilies, and 12 canonical HLA-A and HLA-B supertypes. Thus, alleles grouped in the same supertype have overlapping peptide repertoires with similar peptide-ligand specificity. Therefore, using supertypes significantly reduces data complexity and allows patterns linked to antigen presentation specificity to be found at a deeper level than that of the HLA locus.

Thus, analysis at the supertype level revealed that a higher proportion of ligands derived from translated ncORFs were present in supertype A03 alleles (**Figure 3A**). Similar to the A03 supertype, but with less statistical significance, alleles of the B58 supertype were found to be associated more frequently with proteins derived from ncORFs (**Figure 3B**).

**Figure 3:**
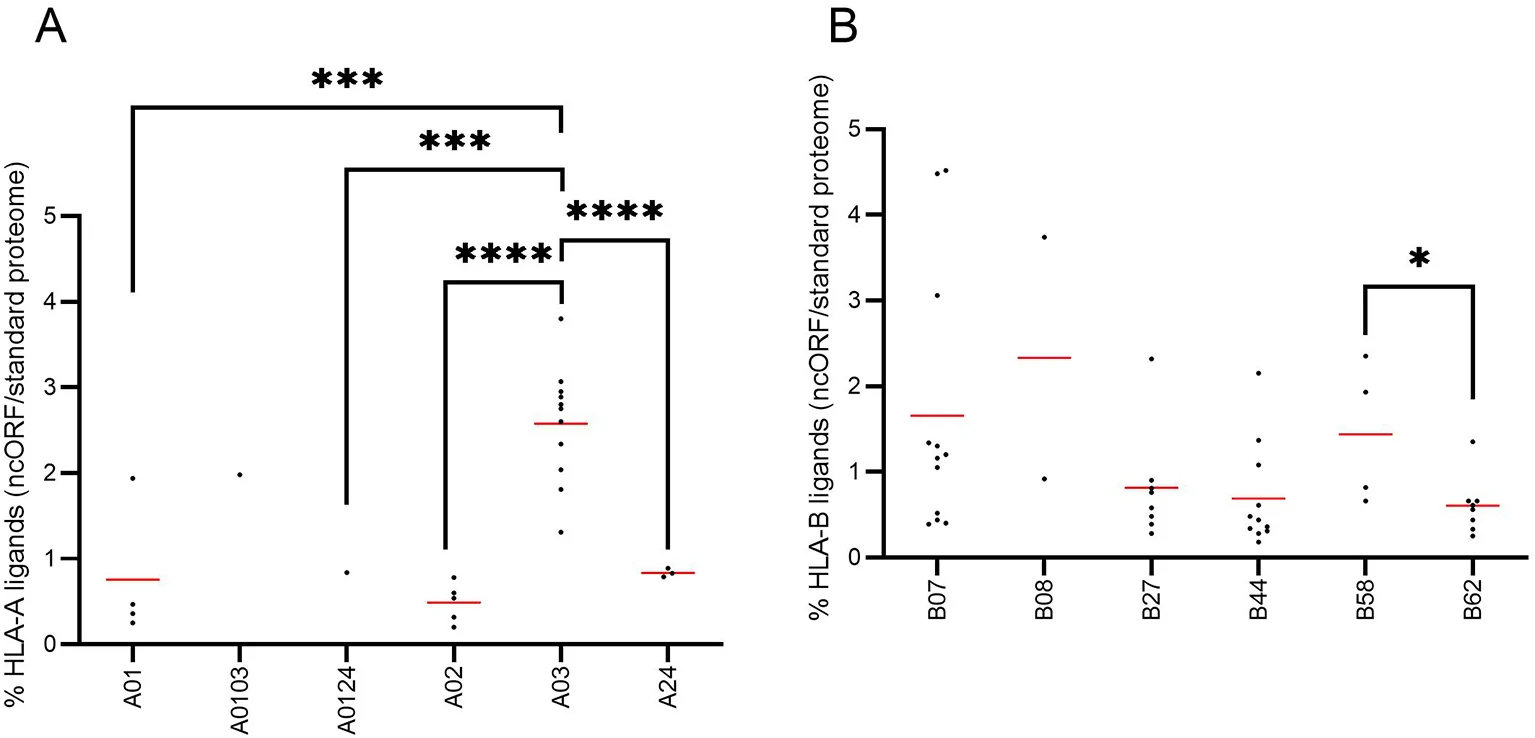
Proportion of HLA class I ligands identified in the 7, 264 ncORFs compared to the standard proteome by HLA-A and -B supertypes. Percentage of HLA class I peptide ligands (ncORFs/standard proteome) presented by each of the HLA-A **(A)** and -B **(B)** class I molecules grouped by HLA supertypes. Statistical significance is shown as follows: p < 0.05, *; p < 0.001, ***; and p < 0.0001, ****.

Overall, these results identify the A03 supertype, and to a lesser extent some HLA-B alleles, as HLA class I groups are most associated with the presentation of peptides derived from translated ncORFs, suggesting the role of HLA polymorphisms in shaping the representation of the non-canonical proteome within the cellular immunopeptidome.

### 3.6 Peptide ligands with basic residues as anchor motif are overrepresented in the ncORFs ligandome

Even after conducting supertype analysis, the range of non-canonical peptide ligands presented by HLA class molecules within the same supertype remained very broad. For instance, the range in supertype A03 varied from 1.3% to 3.8% (**Figure 3A**), whereas in B07, it varied from 0.39% to 4.5% (**Figure 3B**). Consequently, a more thorough examination of the level of peptide binding motifs is essential.

The strong interaction between specific residues of the antigen recognition site of HLA class I molecules and amino acids used as anchor motifs of the peptide ligand, typically at positions 2 or 3 and the C-terminus, allows for subsequent stabilization and export of the nascent HLA/peptide to the cell membrane. Thus, analysis of the specific anchor residue motifs for each of the 102 class I HLA molecules included in this study was performed.

Structurally, six types of amino acid residues (acids, aromatics, basics, hydrophobics, proline, and polar) can be found as anchor motifs associated with different HLA class I molecules. As shown in **Figure 4A**, peptide ligands with a basic residue as anchor motif were significantly more likely to be associated with class I HLA molecules than those with other amino acids. In addition, peptide ligands with proline residue as anchor motif were statistically significant versus aromatic residues in their presentation by class I HLA molecules. These data did not align with the relative abundance of amino acid residues in the ncORFs proteome: acidic (7.0%), aromatic (6.9%), basic (15.7%), hydrophobic (37.9%), proline (8.8%), and polar (23.8%) residues.

**Figure 4:**
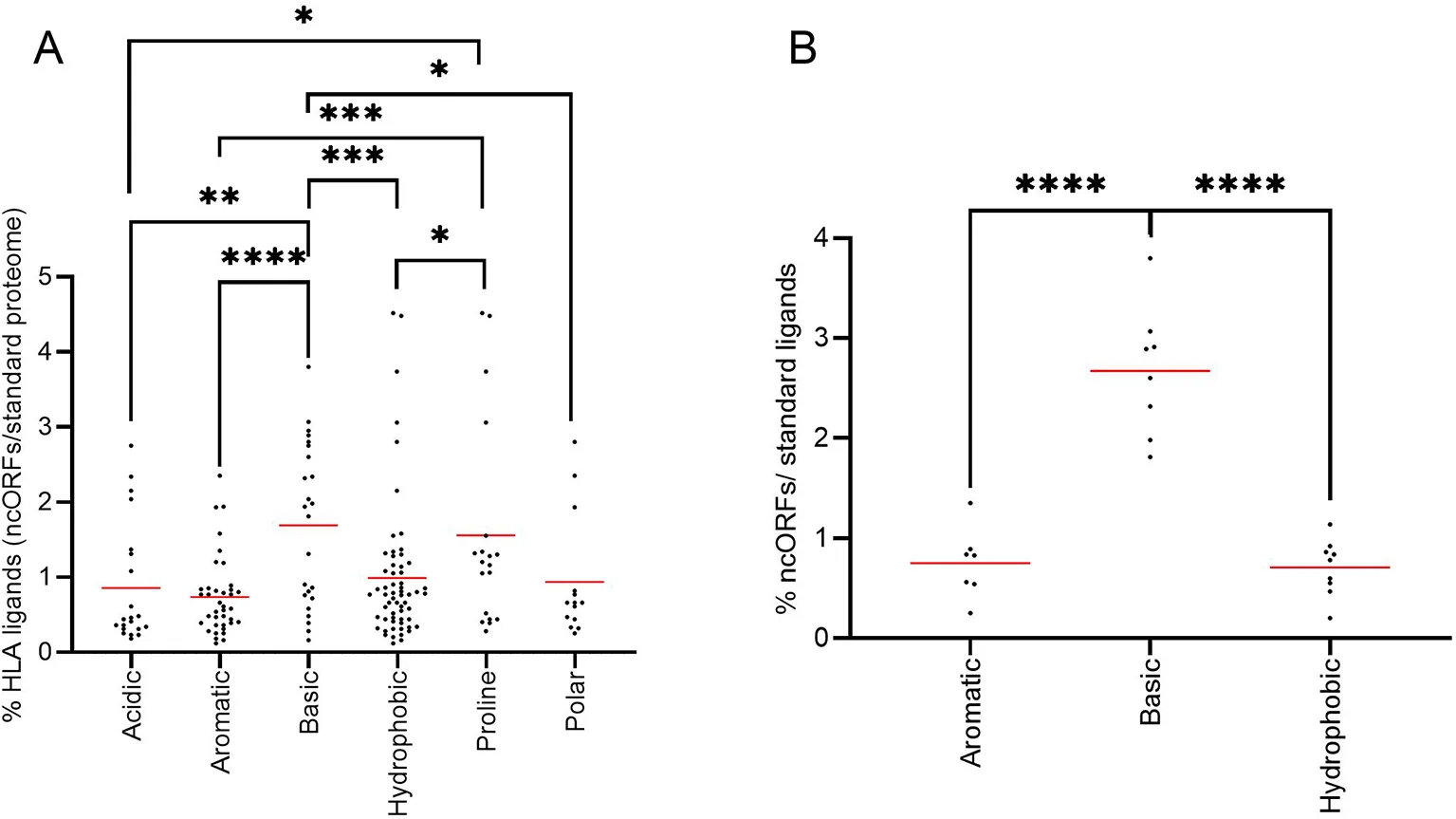
Proportion of HLA class I ligands identified in the 7, 264 ncORFs versus the standard proteome categorized by specific anchor residue motif type. The percentage of HLA class I peptide ligands (ncORFs/standard proteome) presented by each HLA class I molecules grouped by HLA type of specific anchor motif is plotted in A, and the percentage of HLA class I peptide ligands (ncORFs/standard proteome) presented by each of the 24 HLA class I molecules with only aromatic, basic or hydrophobic anchor motifs is plotted in **(B)** Statistical significance is shown as follows: p < 0.05, *; p < 0.01, **; p < 0.001, ***; and p < 0.0001, ****.

Additionally, the same HLA class I molecule can interact with different amino acids in the two major binding motifs (P2/P3 and C-terminus) of the peptide ligand. Therefore, to exclude compensatory effects, an analysis was performed that included only HLA class I molecules with similar structural residues in both anchor motifs. **Figure 4B** shows that the mean ratio of ncORFs/standard ligands with only basic residues as anchor motifs increased from 1.69 ± 1.1 to 2.67 ± 0.64, compared to **Figure 4A**. Similarly, this mean not varied with aromatic residues, but decreased for hydrophobic residues as anchor motifs, dropping from 0.92 ± 0.84 to 0.70 ± 0.28 (**Figures 4A, B**). This improved their statistical significance of the differences compared to basic residues as anchor motifs as shown in **Figure 4A**, reinforcing the preferential association of ncORF-derived peptides with HLA molecules that favour basic anchor motifs.

### 3.7 Dual and triple anchor motifs in the HLA class I polymorphism are relevant in the ncORFs ligandome

Furthermore, peptides bound to the same HLA class I molecule may have different amino acids in the two relevant anchor motifs: the P2/P3 residues and the C-terminal end. Thus, an analysis of these dual anchor motifs was performed for six types of amino acid residues.

The % of HLA ligands from ratio ncORFs/standard proteome was very similar to acidic (0.89 ± 0.82), aromatic (0.73 ± 0.52) and hydrophobic (0.92 ± 0.84) residues (**Figure 4A**). When comparative analysis of acidic residues versus different types of amino acid residues were carried out, acidic residues act as anchor motif at the P2 position in the peptides. As shown in **Figure 5A**, this ratio decreased or remained unaltered when both acidic and aromatic (0.48 ± 0.48) or acidic and hydrophobic (0.74 ± 0.61) residues were present in the same peptide. In contrast, the presence of basic amino acids as C-terminal anchor motif in the peptide sequence increased the identification of peptide ligands with a second acidic anchor motif (**Figure 5A**). As the dispersion of HLA class I molecules data with dual acidic and hydrophobic anchor motifs was high, a more detailed analysis was performed. A comparison between Ala and other hydrophobic residues as C-terminus anchor motif with acidic residues revealed statistical differences in the generation of ligands derived from ncORFs transcripts (**Figure 5A**).

**Figure 5:**
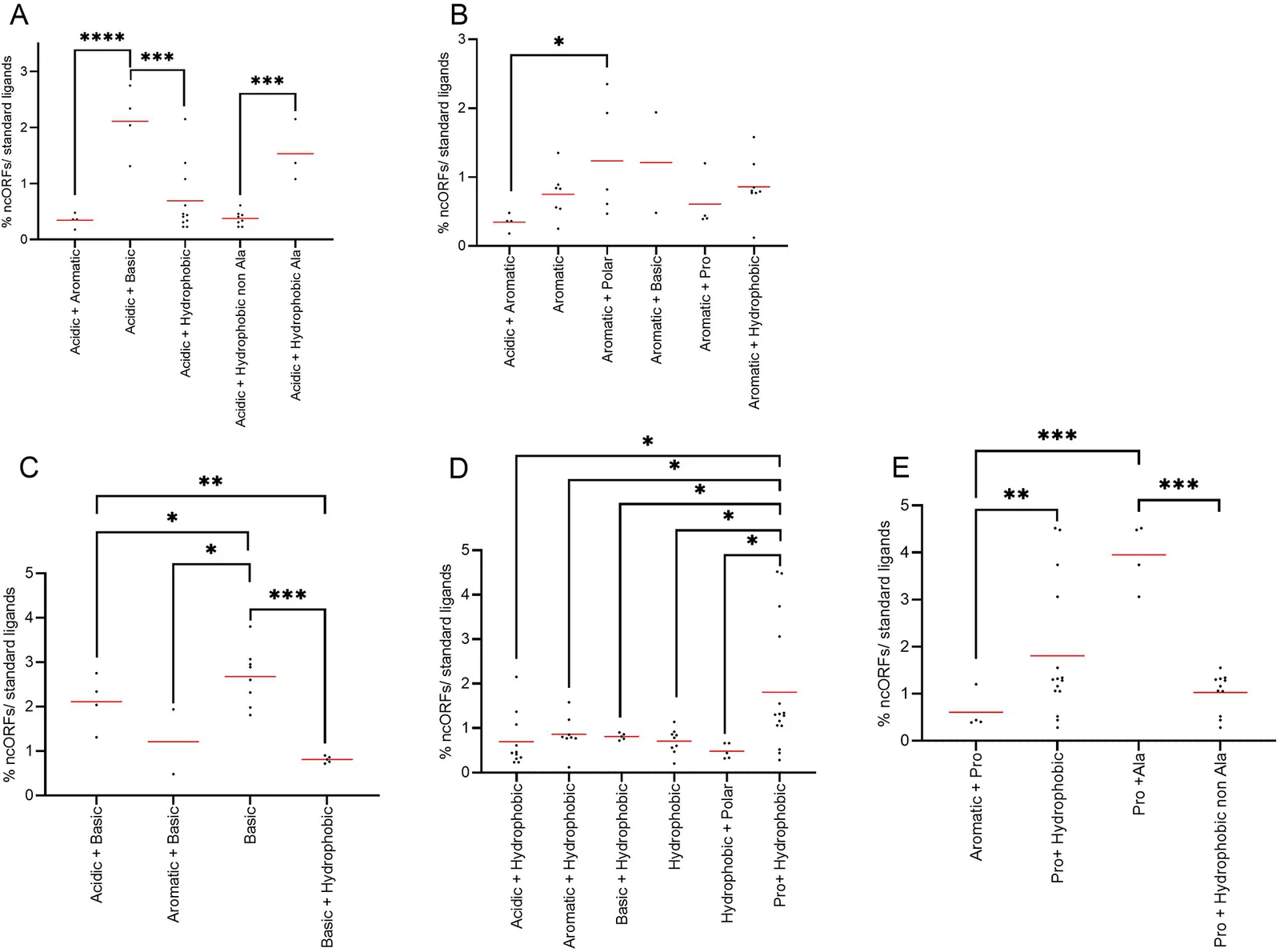
Proportion of HLA class I ligands identified in the 7, 264 ncORFs versus the standard proteome in the 18 HLA class I molecules with different anchor motifs. The percentage of HLA class I peptide ligands (ncORFs/standard proteome) presented by each of the 18 HLA class I molecules with acidic and other amino acid residues as dual anchor motifs **(A)**. The percentage of HLA class I peptide ligands (ncORFs/standard proteome) presented by each of the 25 HLA class I molecules with dual anchor motifs is as follows: aromatic/acidic, aromatic/polar, aromatic/basic, aromatic/Pro or aromatic/hydrophobic **(B)**. The percentage of HLA class I peptide ligands (ncORFs/standard proteome) presented by each of the 13 HLA class I molecules with dual anchor motifs is as follows: acidic/basic, aromatic/basic or basic/hydrophobic **(C)**. The percentage of HLA class I peptide ligands (ncORFs/standard proteome) presented by each of the 47 HLA class I molecules with dual anchor motifs: hydrophobic and acidic, aromatic, basic, polar or Pro residues **(D)**. The percentage of HLA class I peptide ligands (ncORFs/standard proteome) presented by each of the 28 HLA class I molecules with dual anchor motifs: Pro and aromatic or hydrophobic residues **(E)**. Statistical significance is shown as follows: p < 0.05, *; p < 0.01, **; p < 0.001, ***; and p < 0.0001, ****.

In addition, there were no differences in the presence of an aromatic residue and basic, Pro, and hydrophobic amino acids as two binding motifs on the same HLA molecule, whereas the combination of aromatic and polar amino acids versus aromatic and acidic residues was statistically significant (**Figure 5B**). Furthermore, the use of acidic residues as anchor motifs reduced the occurrence of peptides derived from ncORFs transcripts compared with those derived from aromatic residues alone (**Figure 5B**).

A similar analysis with a combination of different residues in one peptide anchor motif and basic amino acids in the other revealed that all combinations (acidic, aromatic and hydrophobic) were statistically different from the basic residues as exclusive anchor motif (**Figure 5C**).

In addition, three different basic amino acids were identified as anchor motif for HLA class I molecules, therefore the individual contributions of these 3 amino acids were analyzed ncORF-derived peptides with Arg and Lys as exclusive anchor motifs were also identified (**Figure 6**). Conversely, His amino acid always acted as an anchor motif in the presence of hydrophobic residues, which served as additional anchor motif (**Figure 6**). The relevance of this basic residue in the generation of ligands derived from unconventional proteins could not be assessed because it was always associated with hydrophobic residues, which significantly reduced the ratio of ligands derived from ncORFs in conjunction with Arg (**Figure 6**). Owing to the high prevalence of basic residues as anchor motifs in peptides, the contribution of these residues at position P2 to the C-terminus was analyzed. The presence of basic amino acids at the C-terminus was statistically significant for the generation of ligands derived from ncORFs (**Figure 6**).

**Figure 6:**
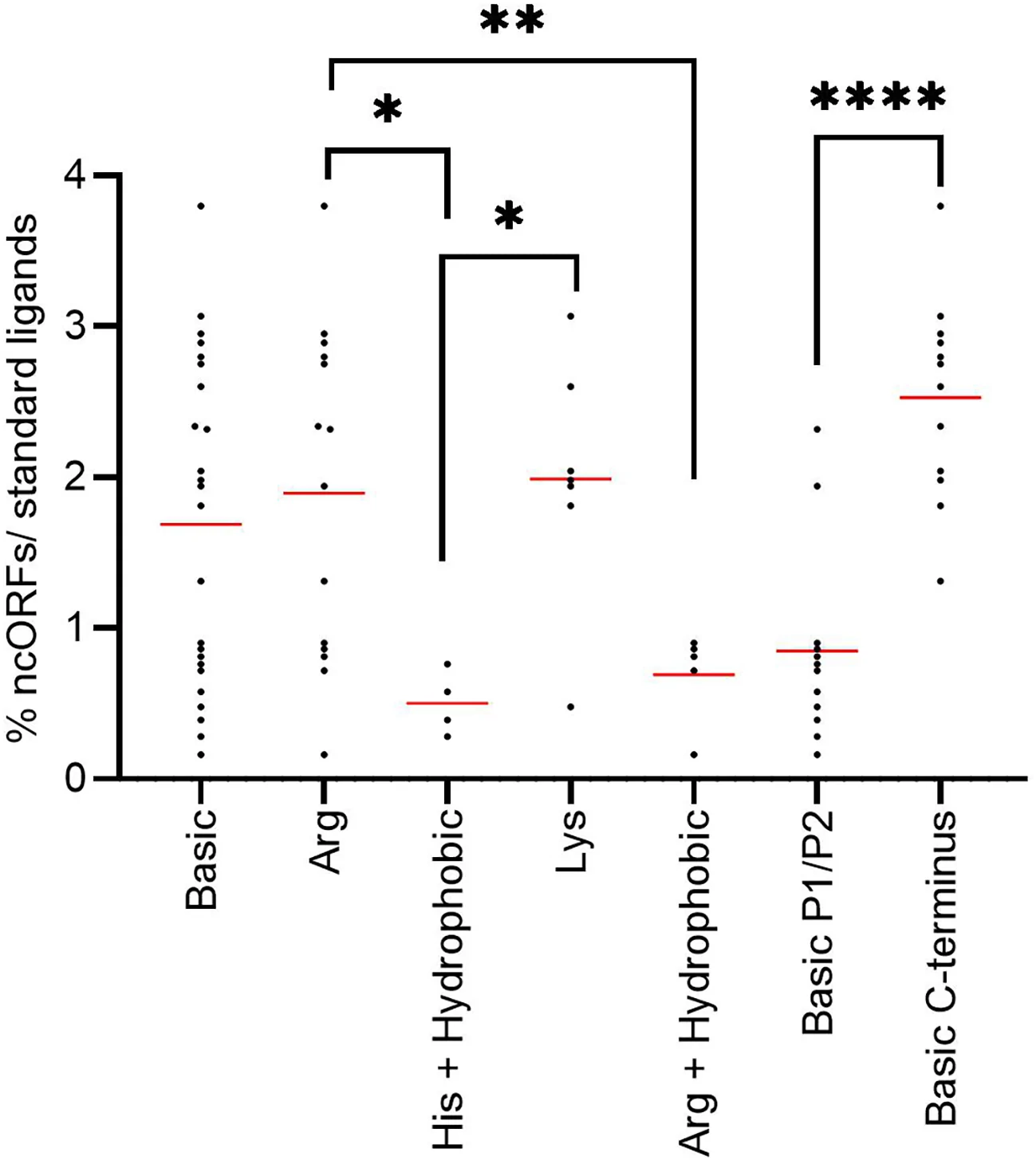
Proportion of HLA class I ligands identified in the 7, 264 ncORFs versus the standard proteome in the 30 HLA class I molecules with specific basic residues. Percentage of HLA class I peptide ligands (ncORFs/standard proteome) presented by each of the 30 HLA class I molecules grouped by a specific basic anchor motif. Statistical significance is shown as follows: p < 0.05, *; p < 0.01, ** and p < 0.0001, ****.

The ncORFs/standard ligands ratio was different for hydrophobic residues as exclusive anchor motif only in combination with Pro amino acids, but not with acidic, aromatic, basic or polar residues (**Figure 5D**). Additionally, those with the highest ratio of Pro and hydrophobic residues contained Ala as amino acids in the C-terminal position.

Finally, Pro acts as an anchor motif at the P2/P3 position in peptides. These HLA ligands always have a second anchor motif at the C-terminus that can be either aromatic or hydrophobic. **Figure 5E** shows that peptides with proline and alanine as dual anchor motifs were statistically overrepresented in the ncORFs ligandome compared to ligands containing proline and aromatic or hydrophobic residues other than alanine. This explains the data dispersion within the B07 supertype (**Figure 3B**), where alleles exhibiting the highest ncORFs/standard ligands ratio were characterized by the presence of Pro and Ala as dual anchor motifs.

Analysis of HLA class I molecules with triple anchor motifs, such as HLA-A*25:01 (which presents ligands with valine or threonine at the P2 peptide position and aromatic residues at the C-terminus) and HLAB*38:01 (which binds ligands with histidine at the P2 position and leucine or phenylalanine at the C-terminus), showed that peptides with basic, polar and hydrophobic anchor motifs were overrepresented in the ncORF-derived ligandome compared with other triple combinations (see **Figure 7**).

**Figure 7:**
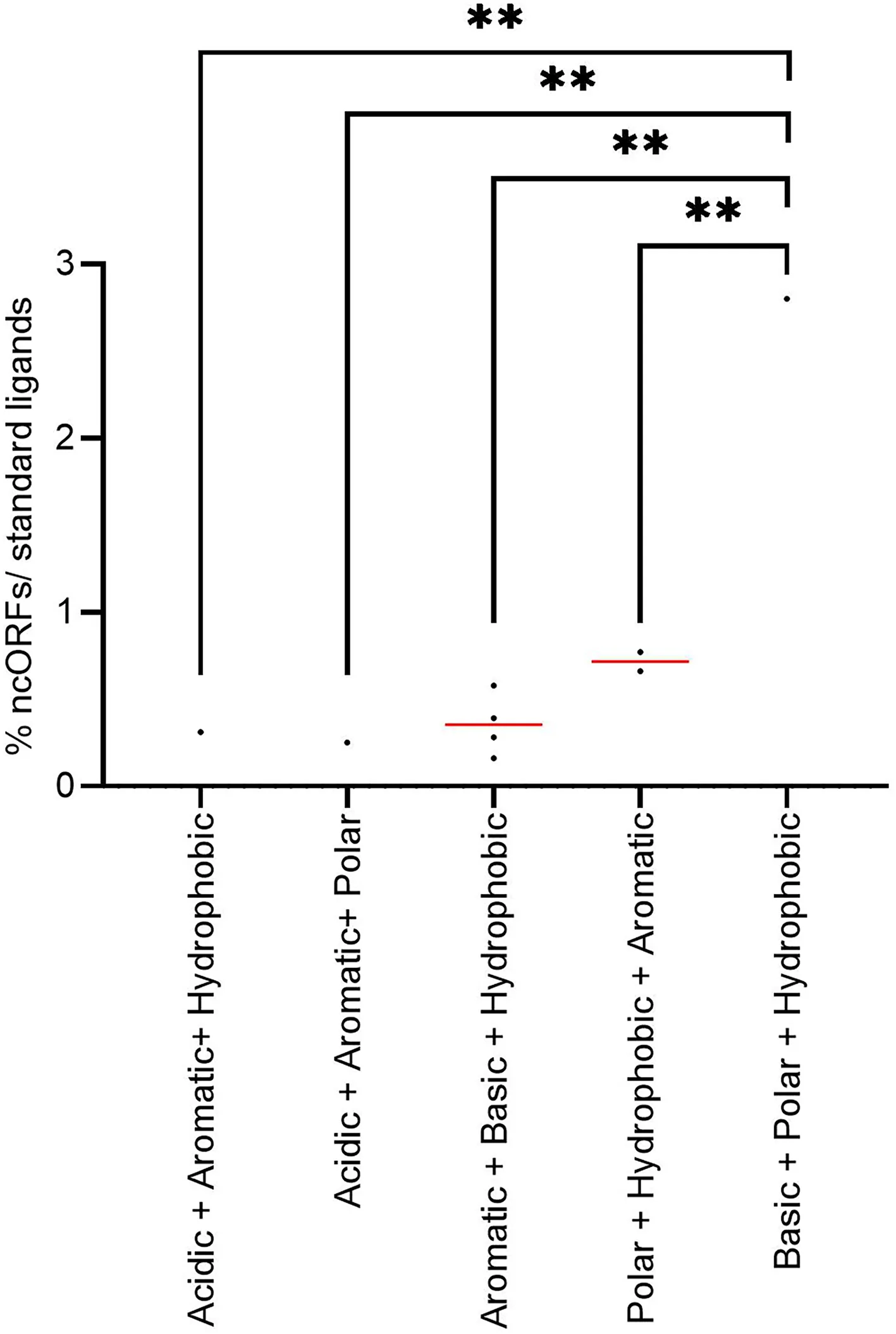
Proportion of HLA class I ligands identified in the 7, 264 ncORFs versus the standard proteome in the 28 HLA class I molecules with triple anchor motif. Percentage of HLA class I peptide ligands (ncORFs/standard proteome) presented by each of the 9 HLA class I molecules with triple anchor motifs. Statistical significance is shown as follows: p < 0.01, **.

Finally, a multiple linear regression analysis incorporating the variables listed in the ‘Materials and Methods’ section to control for potential confounding factors, proved to be a statistically significant global model (*R^2^* = 0.5438) and revealed that the parameters with Anchor Motif Class [basic] (β=1.963, p<0.0001) and [proline + hydrophobic] (β=1.345, p=0.0012) were statistically significant.

## 4 Discussion

In this study, we conducted a detailed analysis of the role of HLA class I polymorphisms in the presentation of peptides derived from this unconventional cellular peptidome. We identified 2, 190 ncORF-derived ligands bound to 102 classical and non-classical HLA class I molecules, with a non-canonical/canonical ratio, mRNA abundance and general characteristics similar to those of other recent analyses (2, 30). We identified, for the first time, ligands derived from translated ncORFs that bind to non-classical HLA-E and -G molecules. All identified non-conventional ligands showed similar theoretical binding affinities and identical anchor motifs for specific HLA class I alleles to those derived from the human conventional proteome, indicating that they are true HLA ligands.

Our findings suggest that HLA polymorphism is associated with differences in the contribution of ncORFs to the HLA class I ligandome. Although ncORF-derived ligands were detected in virtually all analyzed HLA class I molecules, their relative abundances varied markedly among the alleles. The highest proportions of ncORF-derived ligands were observed in several members of the A03 supertype [as was previously reported (31)], as well as in selected HLA-B alleles, particularly those belonging to the B58 supertype. In contrast, the contribution of ncORF-derived products to the HLA class I ligandome was negligible (less than 0.25%) for a variety of HLA class I molecules, including several subtypes of the HLA-A*02 family. These include HLAA*02:01, which is the most prevalent allele in the global population.

A more specific examination at the level of peptide binding motifs of HLA class I molecules revealed that peptide ligands with basic residues as C-terminal anchor motif were overrepresented in the ncORFs ligandome, although Arg, His, and Lys are not particularly abundant in the ncORFs proteome compared to other residues also used as anchor residues for HLA class I binding. Similarly, HLA class I alleles with Pro or acid residues in P2 and Ala, but not other hydrophobic amino acids, in the C-terminus as dual anchor motifs were also overrepresented in the ncORFs ligandome. We currently do not understand the mechanism underlying this differential presentation of ncORF-derived ligands; therefore, further studies are required.

Proteins encoded by ncORFs are promising prognostic markers and therapeutic targets in cancer, as their transcripts are subject to significant dysregulation in various types of tumor tissues (7). In addition, recent studies combining computational prediction, immunopeptidomics, and experimental validation of a limited subset of the candidates have suggested that up to 40% of the tumour-specific antigens in hepatocellular carcinoma are derived from ncORFs (32). Thus, these peptides, which are derived from ncORF-translated products, could serve as ideal targets for CD8^+^ T cell recognition, as demonstrated in an HLA transgenic mouse model (32). Therefore, they can be used in the development of advanced cancer immunotherapy.

In this context, our findings provide a framework for prioritizing candidate antigens for future noncanonical target discovery. Although all the analyzed HLA class I molecules presented ncORF-derived ligands to some extent, nine HLA-A and HLA-B alleles exhibited particularly high proportions of these peptides. Collectively, the cumulative population coverage (carrier frequency) for individuals expressing at least one of these nine alleles is estimated at 36.8% of the global population, as calculated using the IEDB Population Coverage Tool (IEDB Analysis Resource) with average worldwide allele frequencies. Consequently, individuals carrying these alleles represent priority cohorts for the identification and experimental validation of ncORF-derived tumor antigens. Future studies should evaluate whether the enhanced presentation of non-canonical peptides by these HLA molecules correlates with tumour specificity, efficient T-cell recognition and the immunogenic potential of ncORF-derived targets in immunotherapies.

Overall, our results suggest that HLA class I polymorphism is associated with differences in the representation of the non-canonical proteome within the cellular immunopeptidome. These findings broaden our understanding of antigen presentation biology and provide new opportunities for the discovery and clinical exploitation of ncORF-derived tumor antigens.

## Data Availability

Publicly available datasets were analyzed in this study. This data can be found here: Raw MS datasets from the public proteomics repository MassIVE: MSV000084172 (8), MSV000095860 (9) and the PRIDE repository with the ProteomeXchange accession: PXD023064 (10), PXD007679 (11), PXD006215 (12), PXD014804 (13), PXD020186 (14), PXD035241 (15), and PXD009531 (16) were selected.
